# Excessive dietary linoleic acid promotes plasma accumulation of pronociceptive fatty acyl lipid mediators

**DOI:** 10.1038/s41598-022-21823-y

**Published:** 2022-10-25

**Authors:** Nada Birkic, Toni Azar, Krishna Rao Maddipati, Zeljka Minic, Christian A. Reynolds

**Affiliations:** 1 Department of Biotechnology, University of Rijeka, Rijeka, Croatia; 2 Department of Emergency Medicine, Wayne State University School of Medicine, Detroit, MI USA; 3 Department of Pathology, Wayne State University School of Medicine, Detroit, MI USA

**Keywords:** Lipidomics, Risk factors, Pain

## Abstract

Various fatty acyl lipid mediators are derived from dietary polyunsaturated fatty acids (PUFAs) and modulate nociception. The modern diet is rich in linoleic acid, which is associated with nociceptive hypersensitivities and may present a risk factor for developing pain conditions. Although recommendations about fatty acid intake exist for some diseases (e.g. cardiovascular disease), the role of dietary fatty acids in promoting pain disorders is not completely understood. To determine how dietary linoleic acid content influences the accumulation of pro- and anti-nociceptive fatty acyl lipid mediators, we created novel rodent diets using custom triglyceride blends rich in either linoleic acid or oleic acid. We quantified the fatty acyl lipidome in plasma of male and female rats fed these custom diets from the time of weaning through nine weeks of age. Dietary fatty acid composition determined circulating plasma fatty acyl lipidome content. Exposure to a diet rich in linoleic acid was associated with accumulation of linoleic and arachidonic acid-derived pro-nociceptive lipid mediators and reduction of anti-nociceptive lipid mediators derived from the omega-3 PUFAs. Our findings provide mechanistic insights into exaggerated nociceptive hypersensitivity associated with excessive dietary linoleic acid intake and highlight potential biomarkers for pain risk stratification.

## Introduction

Acute and chronic pain are a major cause of suffering and disability worldwide and current therapies aimed at mitigating pain often provide only temporary relief of symptoms^1^. Furthermore, some treatment strategies, including the prescribing of opioids, are associated with risks of developing substance abuse, overdose, and even death^2^. Therefore, identifying modifiable lifestyle factors that influence pain severity are of great value to society.

Nociception is the neural process enabling detection of harmful or potentially harmful stimuli^3^. Nociceptors were first proposed by Sherrington^4^ and refer to receptors that respond to sensory stimuli produced by physiological and pathophysiological stressors (thermal, chemical, or mechanical). Over the past two decades, our understanding of the molecular mechanisms underlying nociception has greatly expanded. The importance of these advances was recently highlighted by awarding of Professors David Julius and Ardem Patapoutian with the 2021 Nobel Prize in Physiology or Medicine for their research into nociception, including the discovery of the transient receptor potential vanilloid 1 (TRPV1) channel^5^. These advancements have helped to identify physiological modifiers of nociception, which will likely usher in novel, targeted therapeutic interventions for acute and chronic pain.

Fatty acyl lipid mediators (LMs) are signaling lipid molecules derived from polyunsaturated fatty acids (PUFAs) and produced by cyclooxygenase (COX), lipoxygenase (LOX) and cytochrome P450 (CYP) enzymes^6^. Many LMs are known mediators of pain and COX inhibitors (e.g., aspirin) are a main strategy to alleviate pain symptoms^7^. However, LMs produced by LOX and CYP enzymes are also involved in nociception^2^. LMs can directly bind ion channels (e.g., TRPV1) located on sensory nerves or indirectly modulate the activation of these neurons via second messenger–signaling pathways, which alter their threshold of activation^8^. The essential PUFAs, alpha linolenic acid (ALA; 18:3n-3) and linoleic acid (LA; 18:2n-6), and their long-chain derivatives, eicosapentaenoic acid (EPA; 20:5n-3), docosahexaenoic acid (DHA; 22:6n-3) and arachidonic acid (AA; 20:4n-6), are the major enzyme substrates for pro- and anti-nociceptive LM production^9^. For example, Resolvin D2, a LM derived from DHA, exhibits potent antinociceptive activity through direct interactions with TRPV1 and transient receptor potential ankyryn 1 (TRPA1) channels^10^. Conversely, the LA-derived epoxide- and dihydroxy- LMs are potent TRPV1 and TRPA1 agonists and exhibit pronociceptive activity^11^.

Human beings are predicted to have evolved consuming a diet relatively low in PUFA and containing a roughly equal ratio of ALA:LA^12^, while the modern diet contains an abundance of LA, which accounts for > 85–90% of dietary PUFA^12,13^. This has led some to hypothesize that dietary LA content may directly influence nociception. Evidence to support this hypothesis was recently published by the laboratory of K.M. Hargeaves. The authors observed that mice maintained on a diet rich in LA displayed nociceptive hypersensitivities when compared to animals maintained on a diet low in LA^14^. Moreover, in patients with chronic daily headaches lowering dietary LA decreased pain severity^15^, and decreases in plasma LA concentrations were associated with clinical pain reduction^16^. Ramsden et al. additionally demonstrated that reduced dietary LA consumption in these patients was associated with reductions in two novel LA derived LMs^17^. However, the extent to which dietary LA content influences the accumulation of other pro- and anti-nociceptive LMs is not completely understood. To test this hypothesis, we used purified triglycerides blends and developed rodent diets rich in either LA or oleic acid (OA; 18:1n-9), a monounsaturated fatty acid that is not metabolized to form LMs. We quantified plasma LMs in male and female rats fed one of the two custom diets from the time of weaning through nine weeks of age. Using this approach, we demonstrate that exposure to a diet rich in LA promotes plasma accumulation of pronociceptive LMs, which likely contributes to the exaggerated nociceptive hypersensitivity associated with excessive dietary LA intake^14,15^.

## Results

Rats were randomized to receive one of two modified AIN-76A rodent diets each containing 5.1% fat. The standard corn oil was replaced with a custom triglyceride blend rich in either LA or OA, a monounsaturated fatty acid that is not metabolized to form LMs. At 9 weeks of age, the rats were euthanized, and plasma samples were collected for fatty acyl lipidomic analysis.

**Figure 1:**
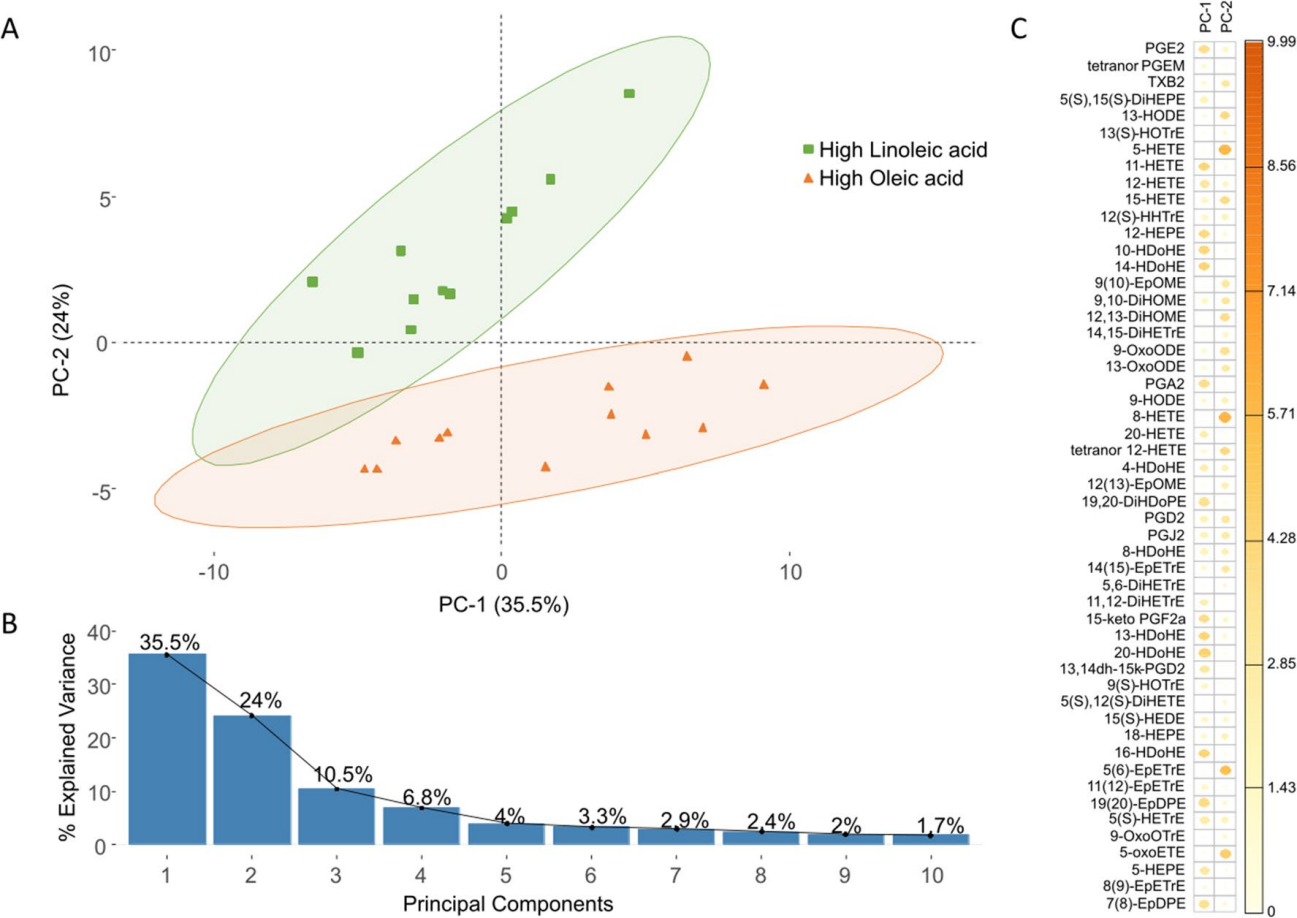
Dietary fatty acid composition determines circulating plasma fatty acyl lipidome content. (**A**) Principal component analysis (PCA) of rat plasma LMs after exposure to a diet rich in LA (green squares, n = 11) or OA (orange triangles, n = 12). Distribution of plasma samples in the space of principle component 1 (PC-1) and principal component 2 (PC-2), which captured 35.5% and 24.0% of the total variance, respectively. Plasma samples were strongly separated along PC-2 based on diet. (**B**) Fraction of total variance explained by PC-1 through PC-10. (**C**) Factor map of individual LMs contributing to PC-1 and PC-2. LMs with larger/darker dots contribute more to the corresponding principal component.

Among female animals, the average body weight at 9 weeks of age in rats fed the LA-rich diet (262.0 ± 25.5 g) was not different than that of rats fed the OA-rich diet (253.3 ± 37.7 g). Male rats maintained on the OA-rich diet tended to be heavier at 9 weeks of age than male rats maintained on the LA-rich diet (376.3 ± 96.5 g vs 338.2 ± 76.1 g), however this difference was not statistically significant. Fatty acyl lipidomic data from all plasma samples (n = 23) were analyzed using principal component analysis (PCA). Figure 1A shows the distribution of plasma samples in the space of principal component 1 (PC-1) and principal component 2 (PC-2), with two clear diet-dependent clusters. PC-1 and PC-2 captured 59.5% of the total variance in the data set (Fig. 1B) and the relative contribution of the individual LM variables to each principal component is shown in Fig. 1C.

**Figure 2:**
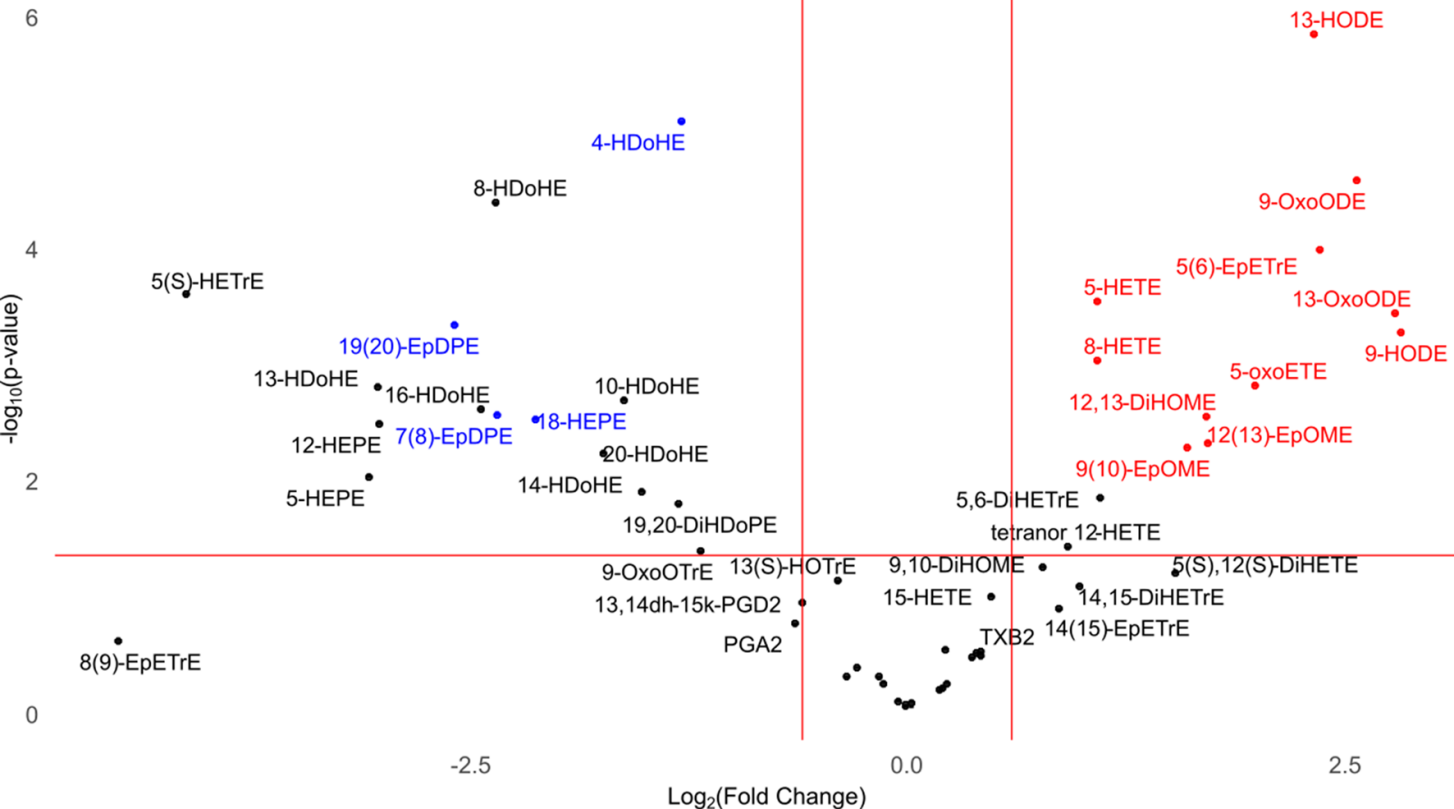
Excessive dietary linoleic acid promotes plasma accumulation of pronociceptive fatty acyl lipid mediators. Volcano plot of rat plasma LMs quantified after exposure to a diet rich in LA (n = 11) vs OA (n = 12). The X axis is the ratio, Log_2_(Fold Change), of the plasma LM concentration in animals fed an LA-rich diet/plasma LM concentration in animals OA-rich diet. LMs with a positive Log_2_(Fold Change) were higher in concentration in the plasma of animals fed the LA-rich diet than in the plasma of animals fed the OA-rich diet. Conversely, LMs with a negative Log_2_(Fold Change) were higher in concentration in the plasma of animals fed the OA-rich diet than in the plasma of animals fed the LA-rich diet. The Y axis (− Log_10_ p value) represents the significance of any differences. LMs with known pronociceptive activity are highlighted in red and those with known antinociceptive activity are highlighted in blue (See Table 1 footnote for the same).

In general, rats maintained on the LA-rich diet displayed greater plasma accumulation of LMs derived from LA and AA, while accumulation of LMs derived from EPA and DHA was greater in rats maintained on the OA-rich diet. Univariate analysis detected 28 LMs with a p-value < 0.05 and a log2fold change > 1.5 (Fig. 2, Table 1), which we classified as “pronociceptive”, “antinociceptive”, or “unknown”, based on a comprehensive search of the MEDLINE database through 28 February 2022 as described in the “Materials and methods”.

**Table 1:** Lipid mediators significantly altered by dietary linoleic acid content.

Precursor PUFA	Fatty acyl lipid mediator	High linoleic acid diet (ng/mL plasma)	High oleic acid diet (ng/mL plasma)	Effect (pro/antinociceptive)	Known mechanism(s)	Reference(s)
Linoleic acid	13-HODE	688.506 ± 283.089	138.452 ± 59.590	**Pronociceptive**	TRPA1; TRPV1; TRPV2; PPARγ	^24–28^
	9-OxoODE	44.765 ± 24.153	7.578 ± 3.038	**Pronociceptive**	TRPV1	^25,26^
	13-OxoODE	80.931 ± 57.003	11.749 ± 5.760	**Pronociceptive**	TRPV1; PPARγ	^25,26,29^
	9-HODE	62.671 ± 46.091	8.915 ± 4.768	**Pronociceptive**	TRPA1; TRPV1; PPARγ	^25–27,29^
	12,13-DiHOME	3.347 ± 2.309	1.026 ± 0.704	**Pronociceptive**	TRPV1; TRPA1	^11,30–33^
	12(13)-EpOME	3.897 ± 2.862	1.184 ± 0.961	**Pronociceptive**	TRPV1; TRPA1	^11,31–33^
	9(10)-EpOME	5.653 ± 4.110	1.868 ± 1.174	**Pronociceptive**	TRPV1; TRPA1	^11,31–33^
Arachidonic acid	5-HETE	17.207 ± 6.048	8.085 ± 4.042	**Pronociceptive**	TRPV1	^15,34^
	8-HETE	7.918 ± 3.227	3.726 ± 1.965	**Pronociceptive**	PPARα; PPARγ	^15,35^
	5-oxoETE	1.264 ± 0.788	0.319 ± 0.444	**Pronociceptive**	Mrgprd	^36,37^
	5,6-DiHETrE	0.095 ± 0.058	0.044 ± 0.032	Unknown		–
	tetranor 12-HETE	9.341 ± 6.457	4.937 ± 2.790	Unknown		–
Eicosapentaenoic acid	5(6)-EpETrE	0.276 ± 0.145	0.054 ± 0.071	**Pronociceptive**	TRPA1; TRPV4	^38,39^
	18-HEPE	0.368 ± 0.457	0.612 ± 1.167	*Antinociceptive*	Unknown	^15^
	12-HEPE	7.709 ± 7.196	62.826 ± 55.451	Unknown		–
	5-HEPE	0.212 ± 0.336	1.796 ± 1.844	Unknown		–
Docosahexaenoic acid	4-HDoHE	1.143 ± 0.592	2.802 ± 0.758	*Antinociceptive*	PPARγ	^40^
	8-HDoHE	0.446 ± 0.316	2.284 ± 1.150	Unknown		–
	19(20)-EpDPE	0.076 ± 0.089	0.459 ± 0.299	*Antinociceptive*	Unknown	^41^
	13-HDoHE	1.166 ± 1.370	9.564 ± 7.671	Unknown		–
	10-HDoHE	1.643 ± 1.240	5.070 ± 3.056	Unknown		–
	16-HDoHE	0.461 ± 0.579	2.509 ± 1.924	Unknown		–
	7(8)-EpDPE	0.011 ± 0.019	0.057 ± 0.041	*Antinociceptive*	Unknown	^41^
	20-HDoHE	0.640 ± 0.500	2.134 ± 1.573	Unknown		–
	14-HDoHE	12.575 ± 10.208	36.203 ± 27.655	Unknown		–
	19,20-DiHDoPE	0.303 ± 0.367	0.753 ± 0.466	Unknown		–
Alpha linolenic acid	9-OxoOTrE	0.183 ± 0.265	0.415 ± 0.261	Unknown		–
Mead acid	5(S)-HETrE	0.253 ± 0.320	4.436 ± 3.172	Unknown		–

LMs with known pronociceptive activity are highlighted in bold and those with known antinociceptive activity are highlighted in italics.

*PUFA* polyunsaturated fatty acid; *13-HODE* (9Z,11E)-13-hydroxyoctadeca-9,11-dienoic acid; *9-OxoODE* (10E,12Z)-9-oxooctadeca-10,12-dienoic acid; *13-OxoODE* (9E,11E)-13-oxooctadeca-9,11-dienoic acid; *9-HODE* (10E,12Z)-9-hydroxyoctadeca-10,12-dienoic acid; *12,13-DiHOME* (Z)-12,13-dihydroxyoctadec-9-enoic acid; *12(13)-EpOME* (Z)-11-(3-pentyloxiran-2-yl)undec-9-enoic acid; *9(10)-EpOME* 8-[3-[(Z)-oct-2-enyl]oxiran-2-yl]octanoic acid; *5-HETE* (5S,6E,8Z,11Z,14Z)-5-hydroxyicosa-6,8,11,14-tetraenoic acid; *8-HETE* (5Z,9E,11Z,14Z)-8-hydroxyicosa-5,9,11,14-tetraenoic acid; *5-oxoETE* (6E,8Z,11Z,14Z)-5-oxoicosa-6,8,11,14-tetraenoic acid; *5,6-DiHETrE* (8Z,11Z,14Z)-5,6-dihydroxyicosa-8,11,14-trienoic acid; *tetranor 12-HETE* (4Z,6E,8S,10Z)-8-hydroxyhexadeca-4,6,10-trienoic acid; *5(6)-EpETrE* 4-[3-[(2Z,5Z,8Z)-tetradeca-2,5,8-trienyl]oxiran-2-yl]butanoic acid; *18-HEPE* (5Z,8Z,11Z,14Z,16E)-18-hydroxyicosa-5,8,11,14,16-pentaenoic acid; 12-HEPE, (5Z,8Z,10E,14Z,17Z)-12-hydroxyicosa-5,8,10,14,17-pentaenoic acid; *5-HEPE* (6E,8Z,11Z,14Z,17Z)-5-hydroxyicosa-6,8,11,14,17-pentaenoic acid; *4-HDoHE* (5E,7Z,10Z,13Z,16Z,19Z)-4-hydroxydocosa-5,7,10,13,16,19-hexaenoic acid; *8-HDoHE* (4Z,6E,10Z,13Z,16Z,19Z)-8-hydroxydocosa-4,6,10,13,16,19-hexaenoic acid; *19(20)-EpDPE* (4Z,7Z,10Z,13Z,16Z)-18-(3-ethyloxiran-2-yl)octadeca-4,7,10,13,16-pentaenoic acid; *13-HDoHE* (4Z,7Z,10Z,14E,16Z,19Z)-13-hydroxydocosa-4,7,10,14,16,19-hexaenoic acid; *10-HDoHE* (4Z,7Z,11E,13Z,16Z,19Z)-10-hydroxydocosa-4,7,11,13,16,19-hexaenoic acid; *16-HDoHE* (4Z,7Z,10Z,13Z,17E,19Z)-16-hydroxydocosa-4,7,10,13,17,19-hexaenoic acid; *7(8)-EpDPE* (Z)-6-[3-[(2Z,5Z,8Z,11Z)-tetradeca-2,5,8,11-tetraenyl]oxiran-2-yl]hex-4-enoic acid; *20-HDoHE* (4Z,7Z,10Z,13Z,16Z,18E)-20-hydroxydocosa-4,7,10,13,16,18-hexaenoic acid; *14-HDoHE* (4Z,7Z,10Z,12E,16Z,19Z)-14-hydroxydocosa-4,7,10,12,16,19-hexaenoic acid; *19,20-DiHDoPE* (4Z,7Z,10Z,13Z,16Z)-19,20-dihydroxydocosa-4,7,10,13,16-pentaenoic acid; *9-OxoOTrE* (10E,12Z,15Z)-9-oxooctadeca-10,12,15-trienoic acid; *5(S)-HETrE* (5S,6E,8Z,11Z)-5-hydroxyicosa-6,8,11-trienoic acid; *TRPA1* transient receptor potential ankyrin 1; *TRPV1* transient receptor potential cation channel subfamily V member 1; *TRPV2* transient receptor potential cation channel subfamily V member 2; *TRPV4* transient receptor potential cation channel subfamily V member 4; *PPARγ* peroxisome proliferator- activated receptor gamma; *PPARα* peroxisome proliferator-activated receptor alpha; *Mrgprd* mas-related G-protein coupled receptor member D.

## Discussion

Here we demonstrate that exposure to a LA-rich diet increases plasma accumulation of pronociceptive LMs. Moreover, exposure to a diet with reduced LA is associated with increased plasma accumulation of antinociceptive LMs derived from EPA and DHA. The latter observation is somewhat unexpected given that these fatty acids were not components of either diet and the concentration of their precursor, ALA, was equal in both diets. It is likely that excess LA interferes with production of antinociceptive LMs derived from the less abundant EPA and DHA. These findings are consistent with finding of Taha et al., which demonstrated that lowering dietary LA in humans increased the plasma concentrations of the omega-3 PUFAs, EPA and DHA.^18^. Excess dietary LA likely competes with ALA for elongation-desaturation enzymes that convert ALA to long-chain omega-3 PUFAs. Additionally, excess dietary LA likely competes with omega-3 PUFAs for enzymes involved in LM production. This further raises the possibility that high LA intake may reduce the benefits of EPA and DHA supplementation on the basis of substrate competition.

The diets used in the present study were manufactured using purified triglycerides, and the only source of dietary PUFA were the essential fatty acids, LA and ALA. This allowed for the dietary fatty acid content to be precisely controlled, beyond what can be done with commercially available oils. An additional strength of this study was the breadth of the panel of LMs quantified, which included most LMs derived from COX, LOX and CYP pathways, for which standards are commercially available. It is important to note that additional novel pro-nociceptive LMs have recently been identified^17^, for which, standards are not commercially available, and thus, these LMs were not quantified in the current study. An additional limitation of our study is the single timepoint of sample collection, which occurred at nine weeks of age. Evidence from humans indicates that the incidence of chronic pain increases with age^19^ and it remains unclear if diet-dependent difference in LM profiles are exaggerated in older rats.

A recent meta-analysis by Li et. al., indicates that higher LA intake is associated with modest reductions in cardiovascular disease risk and mortality^20^. However, as the authors accurately highlight as a major limitation, the default comparison for most of the studies included in their analysis compared high LA intake to high saturated fatty acid intake. It is unclear whether widespread dietary campaigns aimed to reducing cardiovascular disease risk by reducing dietary intake of saturated fat may have inadvertently promoted nociceptive hypersensitivity and/or pain in the population, by increasing LA consumption. Furthermore, given that pain is associated with the development of cardiovascular disease^21^ and nociceptive hypersensitivity may contribute to increasing systemic blood pressure^22^, it remains to be determined whether cardiovascular disease risk could be further reduced if saturated fats were replaced with monounsaturated fatty acids, like OA, which are not substrates for the LM-producing enzymes.

Our findings suggest that dietary LA content may be a modifiable lifestyle factor that can regulate accumulation of pronociceptive LMs and support the notion that LMs can be valuable biomarkers for pain risk stratification. It is tempting to speculate that LMs derived directly from LA, such as the epoxy-octadecenoic acids (EpOMEs) or their corresponding dihydroxy-octadecenoic acids (DiHOMEs) may be particularly attractive biomarker candidates. Further exploration is warranted to determine whether reducing dietary LA content is a viable option for pain management in humans.

## Materials and methods

### Animals and diet exposure

All animal protocols and procedures employed in this study received ethical approval from the Wayne State University Institutional Animal Care and Use Committee (IACUC) and were performed in accordance with the Guide for the Care and Use of Laboratory Animals published by the National Institutes of Health and the ARRIVE guidelines (https://arriveguidelines.org). Male and female littermates (n = 23) from three pregnant Sprague Dawley female rats (Charles River Laboratories, Wilmington, Massachusetts), where used in this study. At the time of weaning, animals were randomized to receive one of two custom AIN-76A rodent diets (Bio-Serv, Flemington, New Jersey) each containing 5.1% fat. The standard corn oil was replaced with one of two custom triglyceride blends. The high linoleic acid oil contained 18.5% tripalmitin (palmitic acid), 18.5% triolein (oleic acid), 60% trilinolein (linoleic acid) and 3% trilinolenin (alpha linolenic acid). The high oleic acid oil contained 18.5% tripalmitin (palmitic acid), 75% triolein (oleic acid), 3.5% trilinolein (linoleic acid), and 3% trilinolenin (alpha linolenic acid). The rats were group housed, maintained on a 12-h light–dark cycle with free access to food and water. All diets were stored at − 20 °C and used within 6 months of manufacturing. At nine weeks of age animals were anesthetized using isoflurane and plasma samples were collected as part of a terminal procedure and stored at − 80 °C until LC–MS analysis was performed.

### LC–MS/MS fatty acyl lipidomic analysis

Fatty acyl lipidomic quantification was performed as previously described^23^ with minor modifications. A complete list of all fatty acyl lipid mediators included in the LC–MS/MS panel, including the IUPAC name, common abbreviation, and link to the pubchem database entry for the compound can be found in Supplementary file 1. Plasma samples (500 μl) were spiked with an internal standard (IS) mix (5 ng each of Prostaglandin E1-d4, Resolvin D2-d5, Leukotriene B4-d4, 15-HETE-d8, and 14(15)-EpETrE-d11) and diluted to 1 mL with 15% methanol in water. Samples were purified on C18 solid-phase extraction cartridges (30 mg sorbent, 1 mL; Strata-X; Phenomenex). The cartridges were preconditioned with 1 ml methanol followed by 1 mL 15% methanol in water. The diluted, IS-spiked samples were applied to the cartridge, washed with 2 mL of 15% methanol and 2 mL hexane, and dried under vacuum for 30 s. The cartridge was eluted with 0.5 ml methanol containing 0.1% formic acid directly into 1.5 mL LC–MS autosampler vials. The eluate was dried under a gentle stream of nitrogen, and the residue was immediately reconstituted with 25 μL methanol. The reconstituted sample was stored at − 80 °C until LC–MS/MS analysis. LC–MS/MS analysis was performed using a C18 column [Luna, C18(2); 2.1 × 150 mm, 3 μm; Phenomenex] and QTrap5500 mass analyzer (AB Sciex) in the negative ion mode. Multiple reaction monitoring (MRM) was used to detect unique molecular ion–daughter ion combinations for each analyte. The data were collected with Analyst 1.7 software (AB Sciex), and the MRM transition chromatograms were quantitated by MultiQuant software (AB Sciex). The IS signals in each chromatogram were used for normalization, recovery, and relative quantitation of each analyte. The concentration of each detected analyte in the plasma samples was expressed as ng per mL.

### Data analysis

Of the LM analytes that were quantified, 53 analytes were detected in > 85% of the samples and were used for subsequent statistical analysis using Rstudio v.4.1.2. Principle component analysis (PCA) was performed using following packages: readxl, FactoMineR, factoextra and corrplot. Unpaired, two-tailed Student’s test was used to generate p-values for use in constructing a volcano plot. Volcano plot analysis was performed using following packages: readxl and ggplot2. Univariate analysis detected 28 LMs with a p-value < 0.05 and a log_2_fold change > 1.5. These LMs were classified as “pronociceptive”, “antinociceptive”, or “unknown”, based on a comprehensive search of MEDLINE database through 20 June 2022. The computer-based search combined search terms related to the individual LM, including the IUPAC name and any synonyms listed on PubChem (https://pubchem.ncbi.nlm.nih.gov/) [e.g. “*(Z)-12,13-dihydroxyoctadec-9-enoic acid*” or “*12,13-dihydroxy-9Z-octadecenoic acid*” or “*12,13-DiHOME*”], and terms related to nociception, including “*nociception*”, “*pain*”, “*allodynia*”, “*algesia*”, “*hyperalgesia*”, “*analgesia*”, and “*hypersensitivity*”. Twenty-two studies were identified reporting on 15 of the 28 LMs that were searched.

## Supplementary Information

Supplementary Information 1.

Supplementary Information 2.

Supplementary Information 3.

## Data Availability

All data generated or analyzed during this study are included in this published article and its supplementary files.
